# Clinical features of hypertensive patients with COVID-19 compared with a normotensive group: Single-center experience in China

**DOI:** 10.1515/med-2021-0225

**Published:** 2021-03-03

**Authors:** Shuang Wang, Qiang Zhang, Peng Wang, Huahong Ye, Xiaoqing Jing, Zhongdan Zhang, Shisheng Zhu, Tingting Luo, Zhaobin Zheng

**Affiliations:** Department of Geriatrics, Changshou people’s Hospital, Chongqing 401220, China; Department of Gastroenterology, Changshou people’s Hospital, Chongqing 401220, China; Department of Nephrology and Hematology, Changshou people’s Hospital, Chongqing 401220, China; Department of Infectious Diseases, Changshou people’s Hospital, Chongqing 401220, China; Department of Cardiovascular, Changshou people’s Hospital, Chongqing 401220, China; Department of Basic Medical Sciences, Chongqing Medical and Pharmaceutical College, Chongqing 401331, China; Department of Gynaecology, Three Gorges Hospital, Chongqing 404100, China

**Keywords:** COVID-19, hypertension, clinical features, ACEI/ARB

## Abstract

**Background:**

SARS-CoV-2 has spread worldwide and poses a great threat to human health. Among COVID-19 patients, those with hypertension have been reported to have higher morbidity and mortality. This study was conducted to provide the international community with a deeper understanding of COVID-19 with hypertension.

**Methods:**

A total of 623 COVID-19 patients enrolled in Wuhan’s hospital were studied from January to March 2020. The epidemiology, clinical features, and laboratory data of hypertensive patients with COVID-19 were collected, retrospectively analyzed, and compared with a normotensive group. The use of antihypertensive drugs, general treatment, and clinical outcomes of hypertensive patients were also analyzed.

**Results:**

The median ages in hypertensive patients with mild and severe COVID-19 were both significantly greater than the median age in the normotensive group. But there was no significant gender difference between the hypertensive and normotensive groups. All patients had lived in Wuhan area. Common symptoms of all patients included fever, cough, and fatigue. Chest computed tomography (CT) scans showed bilateral patchy shadows or ground glass opacity in the lungs of all patients. All (315 (100%)) of the hypertensive patients received antiviral therapy (Umifenovir was used alone or in combination with Ribavirin), antibiotic therapy (215 (68.3%)), and corticosteroids (118 (37.5%)). The results suggest that the combination of Umifenovir and Ribavirin as initial therapy for hypertensive patients with COVID-19 is effective and safe. There were no significant differences in laboratory data between the mild cases in the hypertensive and the normotensive groups. In the severe cases, the hypertensive patients had higher plasma levels of D-dimer, C-reactive protein (CRP), and Interleukin-6 (IL-6) (*P* < 0.05). Furthermore, the hypertensive patients who were treated with angiotensin-converting enzyme inhibitors/angiotensin receptor blockers (ACEI/ARB) were not represented in a statistically significant manner between the mild and severe groups (*p* > 0.05).

**Conclusion:**

In this study, we demonstrated that the hypertensive patients who were treated with ACEI/ARB did not have an increased risk of developing severe COVID-19. Umifenovir and Ribavirin played an important role in the treatment of viral pneumonia. Hypertensive patients with severe viral pneumonia had stronger inflammatory responses than nonhypertensive patients.

## 1 Background

The pandemic of severe acute respiratory syndrome corona-virus 2 (SARS-CoV-2) is causing a huge survival crisis of mankind. The clinical manifestation of this new coronavirus is referred to as coronavirus disease 2019 (COVID-19) [1]. As of December 3, 2020, around 6,48,45,560 coronavirus disease 2019 (COVID-19) cases and 14,99,355 deaths have been reported [2]. Coronaviruses are enveloped, single-stranded, and positive-sense RNA viruses that can be found in many species including humans, other mammals, and birds. They may cause acute respiratory distress syndrome and multiorgan dysfunction [3,4]. Human pathogenic coronaviruses (severe acute respiratory syndrome coronavirus (SARS-CoV) and SARS-CoV-2) bind to their target cells through angiotensin-converting enzyme 2 (ACE2) receptor, which is expressed by epithelial cells of the lung, intestine, kidney, and blood vessels [5]. The function of this enzyme is to catalyze the conversion of angiotensin II to angiotensin 1–7, a peptide that opposes the pro-inflammatory, pro-oxidative, vasoconstrictive, and fibrotic properties of angiotensin II [6]. Hypertensive patients often show decreased expression of ACE2, which suggests that hypertension may be involved in the pathogenesis of COVID-19 [7]. A literature review was performed, and it was found that there have been no studies regarding the characteristics of hypertensive patients infected with COVID-19. The most common symptoms of the disease are fever, dry cough, and fatigue. Pulmonary imaging has shown multiple ground glass shadows in both lungs. Severe cases have been shown to develop into acute respiratory distress syndrome (ARDS) and other serious complications, eventually leading to multiple organ failure.

The aim of this study is to describe the epidemiological and clinical features, laboratory findings, radiological characteristics, treatment, and outcomes of COVID-19 patients with hypertension. It is hoped that these findings will assist the global community to understand more clearly and treat this new infectious disease.

## 2 Methods

### 2.1 Patients

In late December 2019, several hospitals of Wuhan reported clusters of patients with pneumonia of unknown cause, which was identified as SARS-CoV-2 soon after. Wuhan Central Hospital had received a large number of patients infected with SARS-CoV-2. Generally, the patients at Central Hospital come from all regions of Wuhan, especially the Hankou district. In this study, all consecutive patients with confirmed COVID-19 admitted to the main campus of Wuhan Central Hospital from January to March 2020 were enrolled. All patients with COVID-19 enrolled in this study were diagnosed and admitted in accordance with the guidelines of the National Health Commission of China [8]. These data collected from patients’ documented medical files (e.g., the diagnosed patients with antihypertensive drugs and blood pressure <140/90 mm Hg) upon admission were still identified as “with a history of hypertension.” At the same time, these diagnoses were rechecked by two individual investigators (P. W. and Q. Z.) during data collection, using office systolic blood pressure ≥140 mm Hg and/or diastolic blood pressure values ≥90 mm Hg as the criteria [9]. The exclusion criteria included incomplete medical records, pregnancy, acute lethal organ injury, decompensated or end stage of chronic organ dysfunction.


**Ethics approval and consent to participate:** The study has been approved by the ethics committee of Changshou people’s Hospital, Chongqing, China.
**Consent for publication:** Not applicable.

### 2.2 Data collection

We collected the clinical data of patients using the electronic medical record system (HIS). General information included clinical symptoms, physical signs, chest CT manifestations, treatment measures, complications, and outcomes of hypertensive patients. The patients were divided into mild and severe groups. The mild group had symptoms of fever and respiratory tract symptoms, and imaging showed a little ground glass shadow in both lungs. The severe group had respiratory distress, RR ≥ 30 breaths/minute in a resting state, a mean oxygen saturation of ≤93%, and an arterial blood oxygen partial pressure (PaO_2_)/oxygen concentration (FiO_2_) ≤300 mm Hg.

WBC and LYM samples were quantified by fluorescence flow cytometry (XE-5000; Sysmex, Kobe, Japan). An enzyme-linked immunosorbent assay (ELISA) kit (Beijing Biolab Science and Technology Co. Ltd., Beijing, China) was used to detect the level of IL-6. The detection of CRP was performed using a Hitachi 7600 automatic biochemical analyzer (Hitachi Co., Tokyo, Japan). The D–dimer level in patients was detected via immunoturbidimetry kit (Beckman Coulter Inc., USA).

### 2.3 Coronavirus detection

All of the suspected cases were detected by RT-PCR of throat swab samples, and those who were positive for the coronavirus RNA were identified as confirmed cases. Throat swab samples of the patients were collected, and COVID-19 was detected using a novel coronavirus 2019-nCoV nucleic acid detection kit (fluorescent PCR) (Suzhou Tianlong Biotechnology Co., Ltd., Jiangsu, China). The throat swab samples, primers, fluorescent probes, reaction buffer, and enzyme mixture were prepared in a reaction system according to the recommendation of China National Center For Disease Control, two target genes, including open reading frame1ab (ORF1ab) and nucleocapsid protein (N), and simultaneously amplified and tested during the real-time RT-PCR assay. Target 1 (ORF1ab): forward primer CCCTGTGGGTTTTACACTTAA; reverse primer ACGATTGTGCATCAGCTGA; and the probe 5′-FAM-CCGTCTGCGGTATGTGGAAAGGTTATGG-BHQ1-3′. Target 2 (N): forward primer GGGGAACTTCTCCTGCTAGAAT; reverse primer CAGACATTTTGCTCTCAAGCTG; and the probe 5′-FAM-TTGCTGCTGCTTGACAGATT-TAMRA-3′. Reaction mixture contains 12 μL of reaction buffer, 4 μL of enzyme solution, 4 μL of Probe primers solution, 3 μL of diethyl pyrocarbonate–treated water, and 2 μL of RNA template and then amplified according to the following settings: (1) 50°C, 30 min; (2) 95°C, 10 min; (3) 94°C, 15 s → 50°C, 30 s → 72°C, 30 s, 5 cycles; (4) 94°C, 10 s → 58°C, 30 s; 35 cycles.

### 2.4 Statistical analysis

Continuous variables with skewed variables are presented as the median (interquartile range). Categorical variables are expressed as frequencies (percentages). Student’s *t* test or the Mann–Whitney *U* test was used to compare the normally distributed continuous or categorical characteristics, respectively. Statistical analysis was performed with software SPSS version 20.0 for windows (IBM, Armonk, NY, USA). A *P* value of less than 0.05 was considered to indicate statistical significance. All probabilities are two tailed.

## 3 Results

### 3.1 Clinical characteristics

The enrolled 315 hypertensive patients and 308 nonhypertensive patients were all confirmed infected with COVID-19 using PCR tests of throat swabs. The median ages of hypertensive patients with mild and severe COVID-19 were both significantly higher than the median age of normotensive patients (mild group: 61 years (IQR, 56–67) vs 52 years (IQR, 41–63), *P* = 0.001; severe group: 67 years (IQR, 61–74) vs 60 years (IQR, 57–67), *P* = 0.007, Table 1). In the mild COVID-19 group with hypertension, 68 patients (43.9%) were men, and 87 patients (56.1%) were women, which was not statistically significantly different than in the normotensive group (72 patients (47.4%) were men, and 80 patients (52.6%) were women, *p* = 0.538). In the severe COVID-19 group with hypertension, 72 patients (45%) were men, and 88 patients (55%) were women; this observation was also not significantly different than with the normotensive group (64 patients (41.02%) were men, and 92 patients (58.98%) were women, *p* = 0.476). The characteristics of the hypertensive group versus the nonhypertensive group on admission are provided in Table 1. Compared to the normotensive group, the hypertensive group had lower prevalence of fever and had a higher percentage of comorbidities such as diabetes, cardiovascular disease, and cerebral infarction at presentation.

**Table 1:** Comparison of characteristics and symptoms between the hypertensive and nonhypertensive groups infected with COVID-19

	Hypertension	Normotension	*P* value	Hypertension	Normotension	*P* value
	Mildly ill cases (*n* = 155)	Mildly ill cases (*n* = 152)		Severely ill cases (*n* = 160)	Severely ill cases (*n* = 156)	
Age (years)	61 (56–67)	52 (41–63)	0.001	67 (61–74)	60 (57–67)	0.007
**Sex**						
Men, *n* (%)	68 (43.9%)	72 (47.4%)	0.538	72 (45.0%)	64 (41.02%)	0.476
Women, *n* (%)	87 (56.1%)	80 (52.6%)	0.538	88 (55.0%)	92 (58.98%)	0.476
**Comorbidity**						
Hypertension, *n* (%)	155 (100%)	0	—	160 (100%)	0	—
Diabetes, *n* (%)	61 (39.4%)	43 (28.3%)	0.041	56 (35%)	33 (21.2%)	0.006
Cardiovascular disease, *n* (%)	52 (34.2%)	31 (20.4%)	0.009	55 (34.4%)	29 (18.6%)	0.001
Cerebral infarction, *n* (%)	28 (18.1%)	15 (9.9%)	0.039	26 (16.25%)	12 (7.7%)	0.019
Chronic obstructive pulmonary disease	12 (7.7%)	8 (5.3%)	0.379	14 (8.75%)	9 (5.8%)	0.237
malignancy, *n* (%)	0	2 (1.3%)	—	5 (3.1%)	2 (1.3%)	0.266
**Signs and symptoms**						
Fever, *n* (%)	86 (55.5%)	130 (85.5%)	<0.001	92 (57.5%)	135 (86.5%)	<0.001
**Highest temperature, °C**						
<37.3 *n* (%)	69 (44.5%)	22 (14.5%)	<0.001	68 (42.5%)	21 (13.5%)	<0.001
37.3–38.0 *n* (%)	42 (27.1%)	72 (47.4%)	<0.001	41 (26.5%)	72 (47.4%)	<0.001
38.1–39 *n* (%)	34 (29.7%)	44 (18.9%)	0.158	38 (23.8%)	42 (26.9%)	0.517
>39 *n* (%)	10 (7.1%)	14 (13.2%)	0.368	13 (8.1%)	21 (13.5%)	0.126
Cough, *n* (%)	68 (43.8%)	52 (34.2%)	0.083	51 (31.9%)	62 (39.7%)	0.145
Fatigue, *n* (%)	52 (33.5%)	38 (25.0%)	0.1	28 (17.5%)	17 (10.9%)	0.093
Pharyngalgia, *n* (%)	26 (16.8%)	18 (11.8%)	0.218	17 (10.6%)	9 (5.8%)	0.116
Myalgia, *n* (%)	13 (8.4%)	10 (6.6%)	0.547	8 (5%)	5 (3.2%)	0.422
Sputum production, *n* (%)	7 (4.5%)	4 (2.6%)	0.374	9 (5.6%)	11 (7.1%)	0.603
Dyspnea, *n* (%)	5 (3.2%)	3 (1.9%)	0.491	9 (5.6%)	8 (5.1%)	0.834
Headache, *n* (%)	8 (5.2%)	5 (3.3%)	0.415	4 (2.5%)	3 (1.9%)	0.728
Fear of cold, *n* (%)	4 (2.6%)	2 (1.3%)	0.423	6 (3.8%)	4 (2.6%)	0.547
Anorexia, *n* (%)	4 (2.6%)	2 (1.3%)	0.423	4 (2.5%)	3 (1.9%)	0.728
Diarrhea, *n* (%)	5 (3.2%)	1 (0.6%)	0.104	3 (1.9%)	4 (2.6%)	0.677
Chest tightness, *n* (%)	4 (2.6%)	4 (2.6%)	0.423	3 (1.9%)	4 (2.6%)	0.677
Chest pain, *n* (%)	2 (1.3%)	1 (0.6%)	0.573	4 (2.5%)	3 (1.9%)	0.728
Retching, *n* (%)	3 (1.9%)	2 (1.3%)	0.668	2 (1.3%)	2 (1.3%)	0.980
Palpitation, *n* (%)	4 (2.6%)	2 (1.3%)	0.423	1 (0.6%)	1 (0.6%)	0.986
Hemoptysis, *n* (%)	0	0	—	2 (1.3%)	1 (0.6%)	0.989

**Figure 1:**
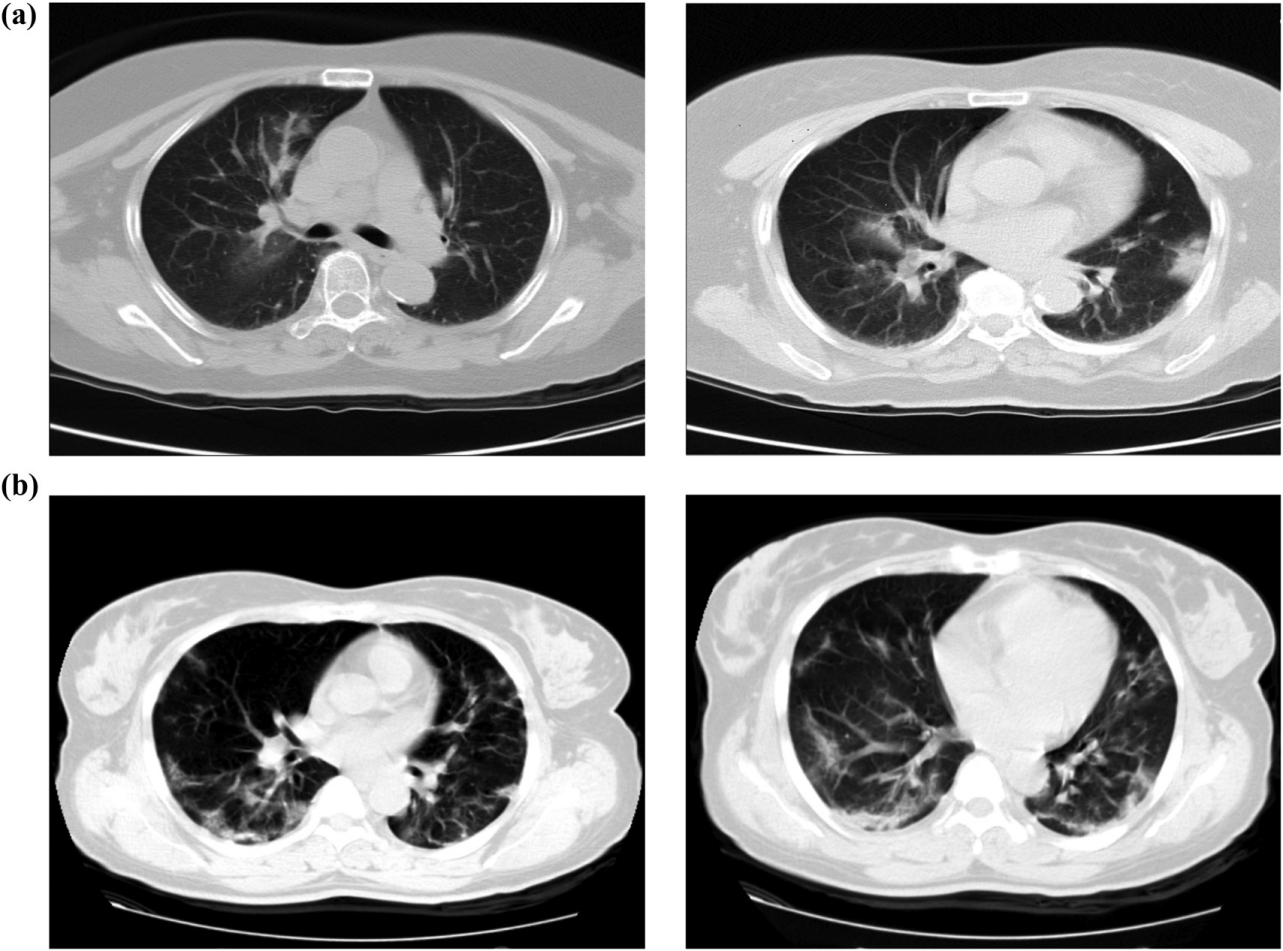
Comparison of laboratory data between the hypertensive and normotensive groups infected with SARS-CoV-2. The level of WBC (a), Lymphocyte (b), CRP (c), PCT (d), IL-6 (e), D–D (f) between hypertension and normotension with different severity of illness.

As shown in Figure 1, there was no difference in the majority of compared laboratory parameters between the mild COVID-19 groups with and without hypertension. However, the levels of CRP, IL-6, and D–dimer in the severe COVID-19 group with hypertension were significantly elevated than in the normotensive severe COVID-19 group (*P* < 0.05).

**Figure 2:**
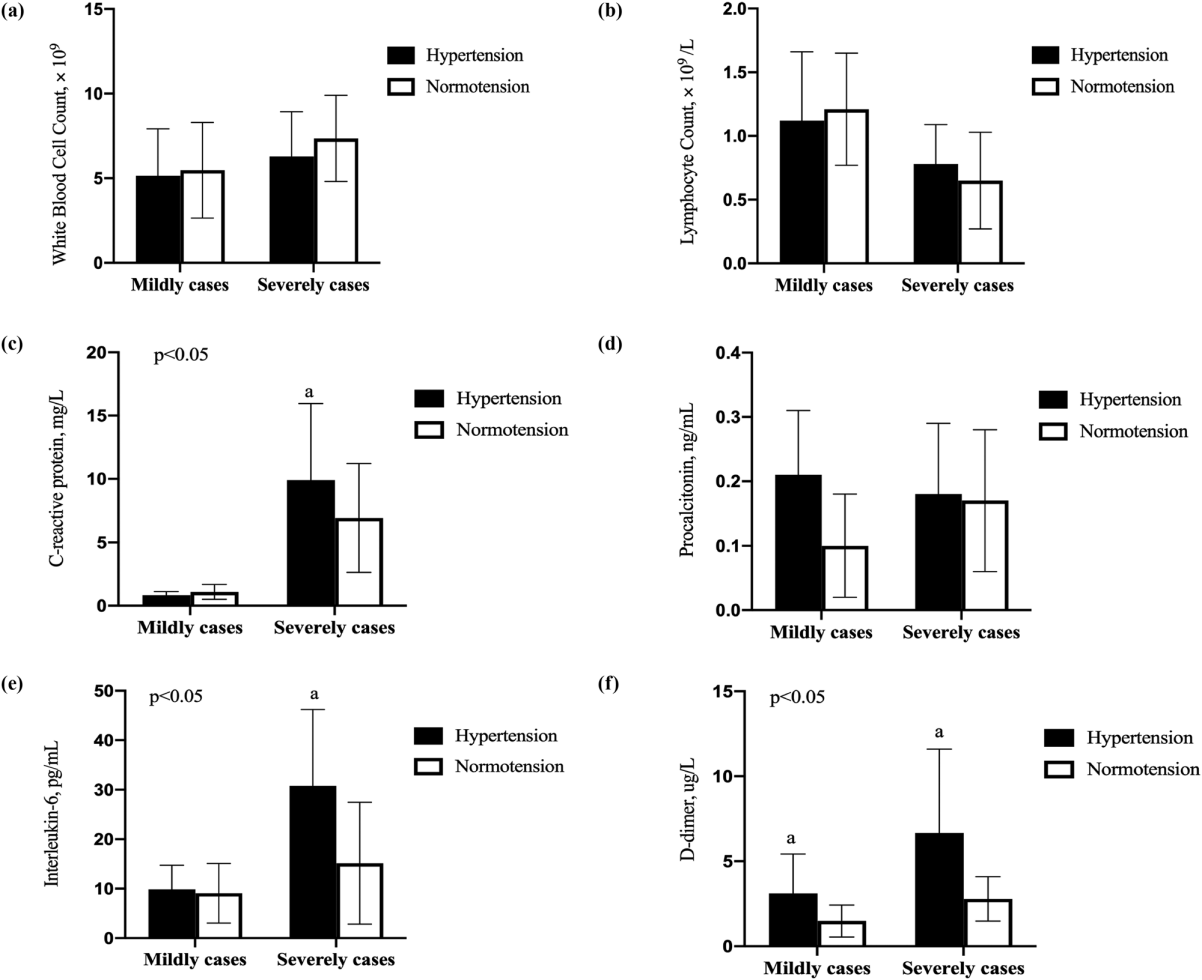
Chest-computed X-ray tomography (CT) images of two patients with COVID-19. (a) Chest CT images of patients in the mildly ill group. (b) Chest CT images of patients in the severely ill group.

All 623 patients had abnormal chest CT findings during admission. The typical characteristics in the imaging showed solitary or multiple patchy shadows and pulmonary interstitial changes with ground-glass opacity and interstitial thickening together with vascular enlargement displaying “slabstone signs.” Two patients’ chest CT images are shown in Figure 2; panel A represents the mild group, and panel B represents the severe group. The opaque area of the ground glass is larger, and the degree of consolidation is more serious in the severe group.

**Figure 3:**
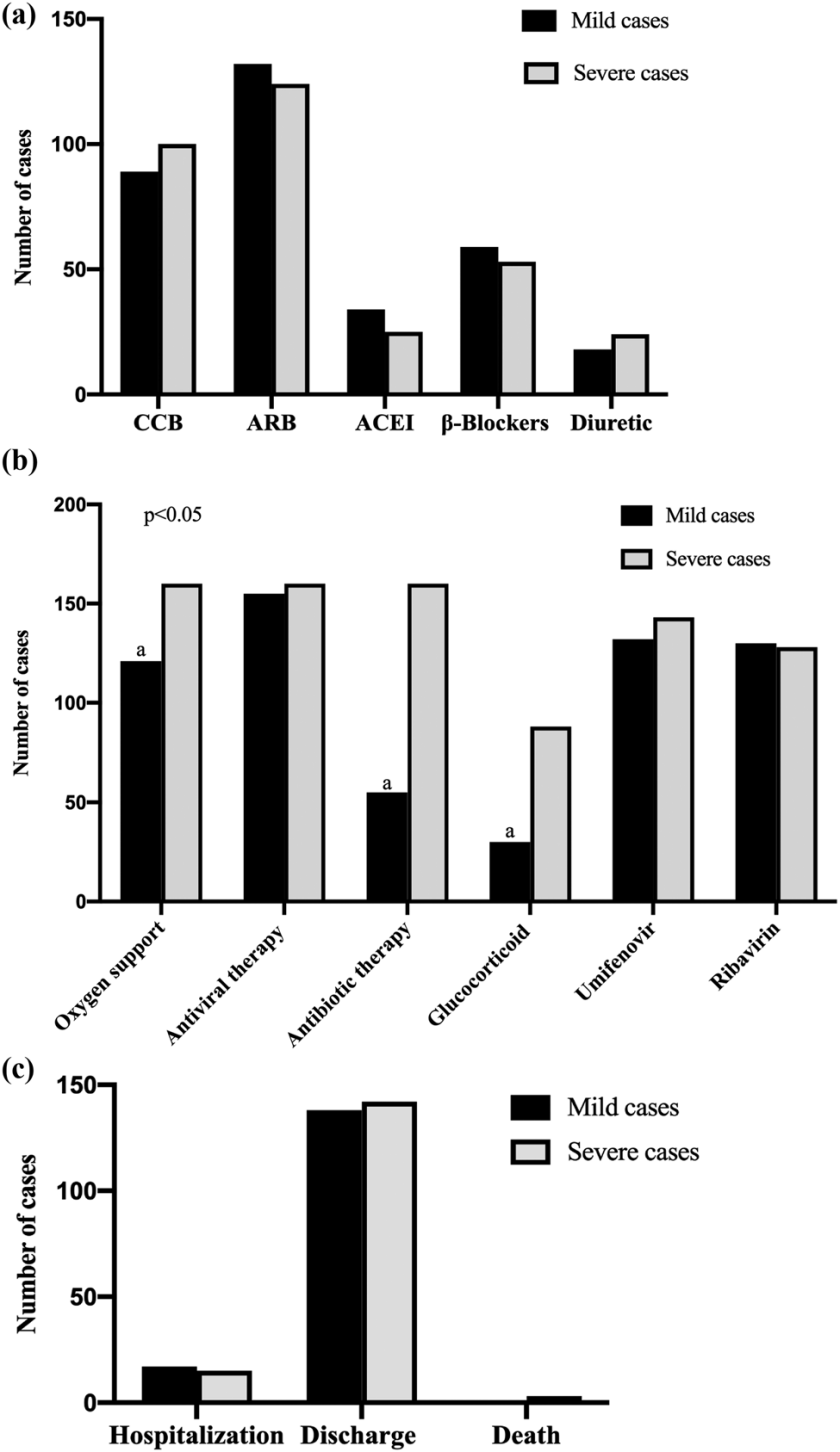
The treatment and outcomes of hypertensive patients with COVID-19. (a) Antihypertensive used in hypertensive patients with COVID-19. (b) General treatment used in hypertensive patients with COVID-19. (c) The outcomes of hypertensive patients with COVID-19. ^a^ *P* values indicate differences between the mild and severe hypertensive patients. *P* < 0.05 was considered statistically significant.

As shown in Figure 3, all of the hypertensive patients received antiviral therapy (315 (100%)), and most of them received antibiotic therapy (251 (68.3%)) and corticosteroids (118 (37.5%)). The majority of patients needed oxygen support (281 (89.2%)). In the hypertensive group, most patients were given calcium-antagonist (CCB, 189 (60%)) and angiotensin-converting enzyme inhibitors/angiotensin receptor blockers (ACEI/ARBs, 59 (18.7%)/256 (81.3%)) to control blood pressure. There was a significant difference between the severe group and the mild group in the number of patients who received antibiotics and corticosteroids (*p* < 0.05). There was no significant difference between the severe and mild COVID-19 groups in the number of patients who used antiviral therapy of Umifenovir (*p* = 0.261) and Ribavirin (*p* = 0.372). At the same time, the two groups (those with severe vs mild COVID-19) showed no statistically significant differences in the numbers of those on antihypertensive therapy taking ACEI/ARBs, CCB, β-blockers, and diuretic medicines (*p* > 0.05). As of March 23, 2020, a total of 280 patients (88.9%) had been discharged, and 3 patients (1%) had died.

## 4 Discussion

Hypertension has been widely reported to increase severity and mortality of patients with COVID-19 [10]. Our research found that the average age of hypertensive patients was over 60 years, which was significantly higher than the average age of patients without hypertension. At the same time, we confirmed that the most common symptoms of hypertensive patients infected with COVID-19 were fever, dry cough, and fatigue. In hypertensive patients, low-grade or moderate fever was common and combined with less systemic symptoms. Some studies have found that ACE2 played a negative regulatory role by degrading Ang II into ang (1–7), which had a protective effect on the lung and could prevent or reduce acute lung failure. SARS-CoV infection decreases the expression level of ACE2, so the protective effect of ACE2 is diminished, making patients more prone to acute lung failure [11]. The expression of ACE2 is significantly decreased in patients with hypertension, which might be one of the reasons why these patients had higher risk of developing severe COVID-19 [12]. But studies have also confirmed that SARS-CoV-2 enters human cells by binding to the ACE2 receptor. Additionally, studies have shown that some antihypertensive drugs such as ACEI and ARBs may increase the expression level of ACE2 at the cell surface [13]. In theory, the susceptibility of patients with hypertension treated with ACEI or ARBs would therefore increase. But research has shown that the acute lung injury caused by the deletion of ACE2 after SARS-CoV infection can be alleviated by reducing the concentration of angiotensin II [14], so the increased expression of ACE2 caused by treatment with ACEI/ARBs may alleviate the lung injury of SARS-CoV-2 to some extent. In our study, there was no significant difference between the mildly ill and severely ill groups taking ACEI/ARBs, so it can be inferred that the ACEI/ARBs can still be taken by hypertensive patients infected with COVID-19.

Research has suggested that SARS-CoV-2 might act mainly on lymphocytes, especially T lymphocytes, just like SARS-CoV [15]. The virus spreads and infects other cells through the respiratory mucosa, induces cytokine storm in vivo, and produces a series of immune reactions, which lead to changes of peripheral white blood cells and immune cells (such as lymphocytes) [16]. IL-6 is an important lymphokine synthesized by T lymphocytes. It directly mediates the responses of the immune system and plays an important role in viral infection and prognosis [17]. In this study, compared with the mildly ill group, the level of IL-6 in the severely ill group increased significantly, as did the level of D–dimer. This also suggested that severely ill patients might have abnormal coagulation. The activation of the coagulation system might be related to the continuous inflammatory response. This may explain why the level of CRP in response to inflammation was also significantly increased. However, PCT is mainly used to reflect systemic bacterial infection. In viral infection or noninfectious inflammation, the level of PCT does not increase or only increases slightly or moderately, which is consistent with the results of this study [18]. In our research, we also found that in the severely ill group, the levels of CRP, IL-6, and D–dimer in hypertensive patients were significantly higher than those in normotensive patients. Disease severity might also be related to ACE2. Hypertensive patients show decreased ACE2 and inhibited degradation of bradykinin (BK). Increased levels of bradykinin and activation of the bradykinin receptor-1 (B1R) by Des‐Arg‐9‐BK can lead to an increase in the secretion of pro-inflammatory cytokines associated with endothelial cell inflammation. IL-6 can be significantly increased with Des‐Arg‐9‐BK stimulation, pointing toward a positive feedback loop in which B1R, activated by pro-inflammatory cytokines, can induce and exacerbate endothelial inflammation [19]. At the same time, prolonged hypertension can easily cause target-organ damage such as damage to the heart, brain, and kidneys. Therefore, in the systemic inflammatory response, the compensatory functions of the important organs of the patients with hypertension decline, and they are unable to deal with the inflammatory storm quickly. But some research also observed that age and comorbidities are the most important determinants of death among COVID-19 patients [20], the average age of hypertensive patients was over 60 years. It is possible that the observed risk may be attributed to the hypertension, together with other comorbidities, the risk influences mortality in older individuals. As such, we need age-adjusted analyses aimed to identify the clinical predictors of severe and fatal COVID-19.

Currently, COVID-19 has no particular effective treatment anywhere in the world. At present, the primary measures to control this disease are early isolation and supportive treatment for the affected patients. In this study, all hypertensive patients were treated with antiviral drugs, Umifenovir and Ribavirin. Ribavirin is a guanosine analog with in vitro activity against a large number of highly lethal emerging viruses. Mechanistically, Ribavirin inhibits RNA synthesis by viral RdRp and also inhibits mRNA capping [21]. According to the results of virtual screening of FDA-approved drugs against the COVID-19 virus main protease (COVID-Mpro), Ribavirin ranked at the second position [22]. Umifenovir is a small indole-derivative molecule. Its mechanism involves inhibition of virus-mediated fusion with target membrane and a resulting block of virus entry into target cells [23,24,29]. In some other studies, Umifenovir has shown a tendency to improve discharge rates and reduce mortality [25]. In our study, the majority of patients were treated with Umifenovir combined with Ribavirin and had higher cure rates (61.2%); so it can be inferred that Umifenovir and Ribavirin are safe and effective in the early treatment of COVID-19 hypertensive patients. Because of high amounts of cytokines induced by SARS-CoV, MERS-CoV [26,27], and COVID-19, in this study, 88 (55%) of the severely ill hypertensive patients were treated with glucocorticoids to reduce inflammatory injury in the lungs. However, the evidence from SARS and MERS indicated that the use of glucocorticoids did not improve mortality, but rather delayed viral clearance [28,29], so the use of glucocorticoids is still controversial. Due to the limited sample size of this study, the efficacy and the safety of glucocorticoids in hypertensive patients should not be assumed proven.

As the sample sizes of hypertensive and nonhypertensive patients were limited, the results of statistical analysis should be interpreted with caution. The *p* values without statistical significance do not necessarily reflect the exact situation of the whole population. Longer-term observation is required.

## 5 Conclusion

COVID-19 is becoming a global health threat, and there is an increasing risk of overwhelming healthcare infrastructures and jeopardizing patient care even in the most developed countries. Research on hypertensive patients infected with COVID-19 can help better determine prognosis and provide a theoretical basis for individualized treatment. Early diagnosis, timely isolation, and appropriate treatment are the keys to fighting this infection.
